# Different Activity Patterns in Retinal Ganglion Cells of TRPM1 and mGluR6 Knockout Mice

**DOI:** 10.1155/2018/2963232

**Published:** 2018-05-08

**Authors:** Haruki Takeuchi, Sho Horie, Satoru Moritoh, Hiroki Matsushima, Tesshu Hori, Yoshitaka Kimori, Katsunori Kitano, Yasuhiro Tsubo, Masao Tachibana, Chieko Koike

**Affiliations:** ^1^Graduate School of Life Sciences, Ritsumeikan University, Kusatsu, Shiga, Japan; ^2^College of Pharmaceutical Sciences, Ritsumeikan University, Kusatsu, Shiga, Japan; ^3^Department of Management and Information Sciences, Faculty of Environmental and Information Sciences, Fukui University of Technology, Fukui, Japan; ^4^College of Information Science and Engineering, Ritsumeikan University, Kusatsu, Shiga, Japan; ^5^Center for Systems Vision Science, Organization of Science and Technology, Ritsumeikan University, Kusatsu, Shiga, Japan; ^6^Precursory Research for Embryonic Science and Technology, Japan Science and Technology Agency, Saitama, Japan

## Abstract

TRPM1, the first member of the melanoma-related transient receptor potential (TRPM) subfamily, is the visual transduction channel downstream of metabotropic glutamate receptor 6 (mGluR6) on retinal ON bipolar cells (BCs). Human TRPM1 mutations are associated with congenital stationary night blindness (CSNB). In both TRPM1 and mGluR6 KO mouse retinas, OFF but not ON BCs respond to light stimulation. Here we report an unexpected difference between TRPM1 knockout (KO) and mGluR6 KO mouse retinas. We used a multielectrode array (MEA) to record spiking in retinal ganglion cells (RGCs). We found spontaneous oscillations in TRPM1 KO retinas, but not in mGluR6 KO retinas. We performed a structural analysis on the synaptic terminals of rod ON BCs. Intriguingly, rod ON BC terminals were significantly smaller in TRPM1 KO retinas than in mGluR6 KO retinas. These data suggest that a deficiency of TRPM1, but not of mGluR6, in rod ON bipolar cells may affect synaptic terminal maturation. We speculate that impaired signaling between rod BCs and AII amacrine cells (ACs) leads to spontaneous oscillations. TRPM1 and mGluR6 are both essential components in the signaling pathway from photoreceptors to ON BC dendrites, yet they differ in their effects on the BC terminal and postsynaptic circuitry.

## 1. Introduction

A fundamental feature of the early vertebrate visual system is the segregation of signals into ON and OFF pathways which separately signal increases and decreases in illumination, respectively. These pathways originate in depolarizing ON bipolar cells (BCs) and hyperpolarizing OFF BCs, which respond with opposite polarity to light-evoked reductions in glutamate release from photoreceptors [1, 2]. In mammalian retinas, rod and cone photoreceptors form synapses with rod and cone BCs, respectively. All rod BCs are ON type, and cone BCs are subdivided into ON and OFF types. The functional diversity of BCs results from the expression of different glutamate receptors (GluRs). ON BCs express a metabotropic GluR, mGluR6, on dendrites that predominantly make invaginating contacts with photoreceptor terminals, whereas OFF BCs express ionotropic GluRs (AMPA/kainate receptors), glutamate-gated cation channels, on dendrites that predominantly make flat contacts at the base of cone photoreceptor terminals [3–5]. In the dark, glutamate released from photoreceptors depolarizes OFF BCs through activation of an ionotropic glutamate receptor, whereas glutamate hyperpolarizes ON BCs through activation of mGluR6 with a decrease in cationic conductance [6–8]. Unlike ionotropic GluRs, which function intrinsically as channels, mGluR6 in ON BCs requires a separate cation channel to mediate visual transduction.

TRPM1, the first member of the melanoma-related transient receptor potential (TRPM) subfamily to be discovered, is the visual transduction channel downstream of mGluR6 on retinal ON BCs [9–12]. Among several splicing variants, a long form of TRPM1 that has six transmembrane domains is specifically expressed in the same ON BCs as mGluR6. In common with mGluR6, human TRPM1 mutations are associated with CSNB [13, 14]. TRPM1 and mGluR6 are both critical for initiating ON response in retinal BCs, and absence of either gene abolishes light responses in retinal ON BCs [15].

Here, we report a striking difference in the firing patterns of retinal ganglion cells (RGCs) between TRPM1 KO and mGluR6 KO mouse retinas. RGCs in the TRPM1 KO retina show spontaneous oscillations whereas those in the WT and mGluR6 KO retinas do not. We investigated the morphology of rod BC terminals in the TRPM1, WT, and mGluR6 KO retinas and found a defect in rod BC terminal shape only in the TRPM1 KO retina. We suggest that the differences in terminal shape and oscillatory behavior are causally related. In this scenario, the lack of appropriate rod BC input to AII ACs leads to spontaneous oscillations.

## 2. Materials and Methods

### 2.1. Animals

We exclusively used 2-3-month-old mice on a 129 Sv/ev background. TRPM1 KO and mGluR6 KO mouse lines were generated by disruption of the TRPM1 or mGluR6 gene by homologous recombination and provided by Drs. Takahisa Furukawa and Shigetada Nakanishi, respectively [7, 12]. All procedures conformed to the ARVO Statement for the Use of Animals in Ophthalmic and Vision Research and were approved by the Institutional Safety Committee on Recombinant DNA Experiments and the Animal Research Committee of Ritsumeikan University. Mice were housed in a temperature-controlled room with a 12 hr light/dark cycle. Fresh water and rodent diet were available at all times.

### 2.2. Electrophysiology

Adult (postnatal day < 28) mice were dark-adapted for more than 1 hr. Mice were euthanized under dim red light and eyes were enucleated. Subsequent procedures were performed under a stereomicroscope equipped with an infrared (IR) image converter (C5100, Hamamatsu Photonics) and IR illumination (HVL-IRM, Sony). After the cornea and lens were removed, a small cut was made at the dorsal part of the eye cup as a landmark. Using the landmark, the ventral retina isolated from the pigment epithelium was reproducibly oriented and positioned on the multielectrode array (60pMEA200/30iR-Ti, MEA2100Lite (Multi Channel Systems, Reutlingen, BW, Germany)) with the GC layer facing down. The retina was continuously superfused with Ames Medium (Sigma-Aldrich, MO, USA) bubbled with 95% O_2_/5% CO_2_ at a rate of 4 mL/min. The superfusate in the recording chamber was maintained at 32 ± 1°C. A light stimulus was presented on a monitor display (FG2421, EIZO, Ishikawa, Japan) and focused onto the retina with optics. The retina was stimulated with a white square (1,600 × 1,600 *μ*m on the retina) for 2 s (187.8 × 10^−3^ cd/m^2^; within the scotopic range [16]) on a dark background (0.1 × 10^−3^ cd/m^2^) every 10 s. Recorded spike discharges were sorted by Efficient Technology of Spike-Sorting (EToS4) [17] and analyzed with custom software. To verify the accuracy of sorting, we performed autocorrelation analysis on the sorted spike train from each unit and confirmed the presence of a refractory period (0 ± 2 ms). Oscillations of spiking were evaluated based on the power spectrum, which was the fast Fourier transform (FFT) of the autocorrelogram. An oscillation index (OI) was defined as (maximum_5–15 Hz_ − mean_5–15 Hz_ )/SD_0.5–500 Hz_ in the power spectrum, and RGCs with OI ≥ 8 were identified as oscillatory, whereas RGCs with OI < 8 were nonoscillatory.

### 2.3. Immunohistochemistry

Mouse retinas were fixed, embedded into agarose, and cut into 50 *μ*m thick sections by Linear Slicer PRO7 (Dosaka EM Co. Ltd., Kyoto, Japan). For immunostaining, we used a mouse monoclonal anti-PKC*α* antibody (P5704, Sigma-Aldrich, MO, USA) and a guinea pig polyclonal anti-VGluT1 antibody (AB5905, Millipore, MA, USA). Sections were imaged with a LSM700 confocal microscope (Carl Zeiss Microscopy, Thüringen, Germany). To quantify the morphology of the rod BC synapse, every 5th frame of a confocal z-stack (interval = 0.275 *μ*m) was collapsed by the maximum intensity projection (MIP) method. The terminal region was segmented by eye and the area and perimeter were measured with ImageJ software (http://rsb.info.nih.gov/ij/). Statistical analyses were performed using GraphPad Prism v7 (GraphPad Software). Multiple comparisons were performed using one-way ANOVA with post hoc Tukey's test. The number of samples analyzed is indicated in the figure legends.

## 3. Results

### 3.1. Spontaneous Oscillatory Activities of RGCs in the TRPM1 KO Mouse Retina

TRPM1 and mGluR6 cooperatively regulate the visual cascade in ON BCs and both are associated with CSNB. In both TRPM1 and mGluR6 KO mouse retinas, retinal ON BCs lacked ON responses but OFF BCs generated OFF responses. We then tested whether both knockouts had similar effects on RGC responses to light stimulation.

**Figure 1:**
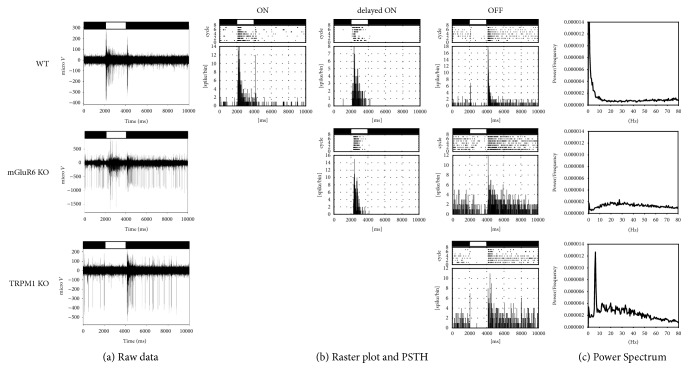
RGCs in TRPM1 KO but not in mGluR6 KO retinas showed obvious oscillations during MEA recordings. Light-evoked responses were recorded from the wild type (WT), mGluR6 KO, and TRPM1 KO RCGs. (a) Example traces of firing during a 2-s light stimulus (light/dark bar at the top of each panel). Data were obtained from the WT* (upper panel)*, mGluR6 KO* (middle panel)*, and TRPM1 KO* (lower panel)* retinas. Traces show the responses of one or more RGCs recorded on a single electrode. (b) Raster plots of spikes sorted from the firing in (a) and their resulting peristimulus time histograms (PSTHs; 5-ms bin width). In these examples, spikes were sorted into three (sustained ON, delayed ON, and transient OFF) RGCs in WT* (upper)*, two (delayed ON and sustained OFF) RGCs in mGluR6 KO* (middle)*, and one (sustained OFF) RGC in TRPM1 KO* (lower)*. (c) Power spectra of firing calculated from the OFF RGCs shown in (b) (*upper*, WT;* middle*, mGluR6 KO; and* lower*, TRPM1 KO). A peak (~8 Hz) was detected only in TRPM1 KO.

**Figure 2:**
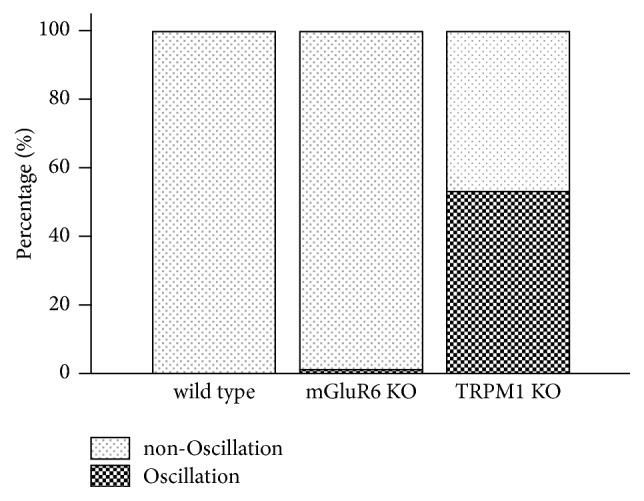
The percentages of oscillatory RGCs in WT (left), mGluR6 KO (middle), and TRPM1 KO (right). None of 173 RGCs (3 WTs), 2 of 152 RGCs (3 mGluR6 Kos), or 129 of 242 RGCs (3 TRPM1 KOs) showed obvious oscillations (OI ≥ 8, see* Methods and Materials*).

We recorded extracellular RGC spike discharges in whole-mounts from the TRPM1 KO retina using a MEA and compared them with those from the wild type (WT) and mGluR6 KO retinas. Unexpectedly, we observed prominent oscillatory firing in the TRPM1 KO retina, but not in the WT or mGluR6 KO retina (Figure 1). Example traces of firing during a 2-s light step were obtained from the WT, mGluR6 KO, and TRPM1 KO retinas (Figure 1(a)). Spike discharges recorded from each electrode (Figure 1(a)) were sorted into clusters, and the firing of each cluster was analyzed separately in raster plots and peristimulus time histograms (PSTHs) (Figure 1(b)). In these examples, spikes were sorted into three (sustained ON, delayed ON, and transient OFF) groups in WT, two (delayed ON and sustained OFF) in mGluR6 KO, and one (sustained OFF) in TRPM1 KO (Figure 1(b)). We detected long-latency ON responses (delayed ON) in the WT and mGluR6 KO retinas as previously reported (Figure 1(b)) [18, 19]. Power spectra were obtained from the discharges of OFF RGCs from each genotype. A prominent peak (~8 Hz) was detected only in the TRPM1 KO, but not in the WT and mGluR6 KO retinas (Figure 1(c)). The fraction of oscillatory RGCs was more than 50% in the TRPM1 KO but almost zero in the WT and mGluR6 KOs (Figure 2). Thus, the TRPM1 KO retina contains an intrinsic oscillatory circuit that is absent from the WT and mGluR6 KO retinas.

### 3.2. The Synaptic Terminals of Rod BCs Are Significantly Smaller in the TRPM1 KO Retina

**Figure 3:**
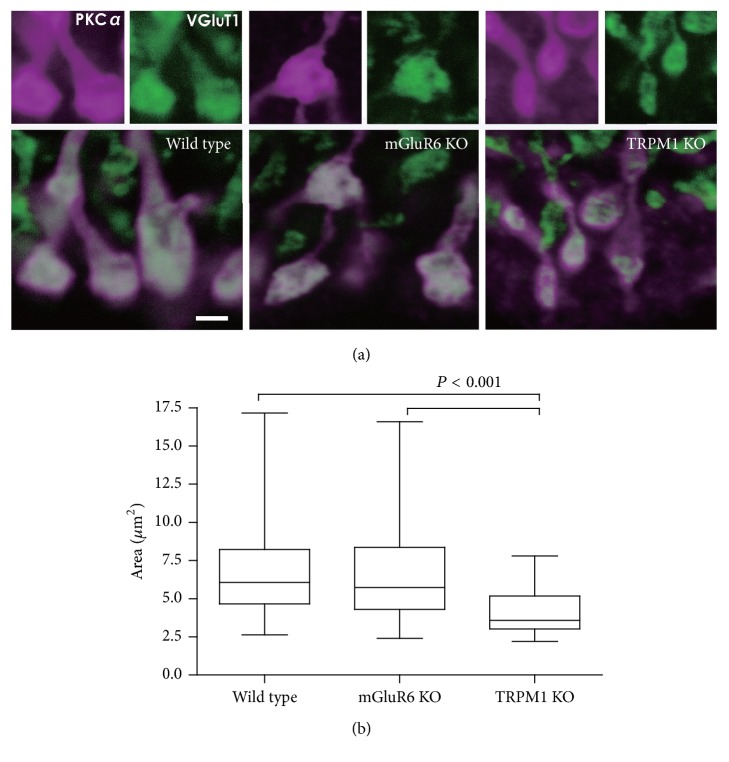
Immunohistochemical labeling of WT, mGluR6 KO, and TRPM1 KO retinas. (a) Rod BC terminals in the TRPM1 KO retina* (right)* were smaller in size than those in WT* (left)* and mGluR6 KO* (middle)* retinas. PKC*α* (magenta) and VGluT1 (green) staining. Scale bar, 2 *μ*m. (b) Size (area) of PKC*α*-stained synaptic terminals of rod BCs. Terminal size in the TRPM1 KO retina was significantly smaller than that in WT and mGluR6 KO retinas (*P* < 0.001 by one-way ANOVA, post hoc Tukey's test). Error bars represent the SD from the mean of 134 (3 WTs), 115 (3 mGluR6 KOs), and 45 (3 TRPM1 KOs) terminals.

To understand the intrinsic mechanisms of oscillations in the TRPM1 KO retina, we investigated the synaptic terminal morphologies of retinal ON BCs. We identified rod BCs by immunohistochemistry using an anti-PKC*α* antibody (Figure 3(a)). The synaptic terminal areas of the TRPM1 KO retina were significantly smaller than those of the WT retina (Figure 3(b)), as reported by Kozuka et al. (2017) [20]. Synaptic terminals in the mGluR6 KO retina were almost identical to those in the WT retina and also significantly different from those in the TRPM1 KO retina. Similar differences in terminal size were observed when VGluT1 was used as a terminal marker (Figure 3(a)).

## 4. Discussion

We have shown that the pattern of RGC activity differs between TRPM1 and mGluR6 KO retinas. (1) Namely, oscillations were observed in the TRPM1 KO retina but not in the mGluR6 KO retina (Figures 1 and 2). (2) In addition, rod BC terminal size was significantly smaller in the TRPM1 KO retina when compared to the WT or mGluR6 KO retina (Figure 3), in agreement with a recent report [20].

In rd1 and rd10 mice, a loss of distal retinal input leads to aberrant oscillatory activity in RGCs in association with abnormal function in the AII AC-ON cone BC network [21–24]. Although oscillatory frequencies in the remaining network are higher in the rd1 retina (10–16 Hz) than the rd10 retina (3–7 Hz), the origin of rhythmic activities seems to be similar in both cases [25]. Two distinct mechanisms of spontaneous oscillations have been described: one arises from the outer retina and the other from the inner retina [26]. The maximum frequency of oscillation in the outer retinal is 3 Hz whereas that in inner retinal cells is higher (ON cone BCs: 15.1 Hz; AII ACs: 16.8 Hz; ON RGCs: 10.6 Hz; OFF RGCs: 5.5 Hz; ON-OFF RGCs: 7.1 Hz) [21, 26, 27]. The oscillatory frequency of RGCs in the TRPM1 KO retina is ~8 Hz (Figure 1(c)), within the range of frequencies displayed by inner retinal neurons in rd mice. In rd mice, inner retinal oscillations are thought to originate in the AII AC-ON cone BC network [27] or in the intrinsic bursting of AII ACs [28, 29]. Computational modeling studies suggest that AII AC hyperpolarization is a common precondition for bursting and oscillations in the AII AC-cone BC network [27, 29] or AII AC itself [26]. Rod BC terminals are smaller but the outer retina is intact in the TRPM1 KO retina, whereas rod BC terminals are not obviously changed but the outer retina is severely degenerated in the rd1 and rd10 retinas [21, 30]. Although the affected retinal location is different among these mutant models, they share common alternations, such as structural changes and reduction of visual input which may reduce the drive through rod BCs to the AII AC-cone BC network. Therefore, the origin of rhythmic activities would be shared among TRPM1 KO and rd retinas.

Based on the idea that AII AC hyperpolarization is a precondition for oscillations, we propose three potential mechanisms through which a loss of TRPM1 channels could lead to AII AC hyperpolarization: first, rod BCs are hyperpolarized and release less glutamate onto AII ACs at their terminals; second, small rod BC terminals release less glutamate onto AII ACs; and third, developmental abnormalities related to the first and second points lead to a hyperpolarized state in AII ACs. These mechanisms, which are not mutually exclusive, will be examined below.

Differences in rod BC resting membrane potential in the TRPM1 and mGluR6 KO retinas could be due to the different functions of these proteins in ON BC transduction: TRPM1 is a nonselective cation channel whereas mGluR6 is a regulator. In this view, a lack of mGluR6 in the KO leads to disinhibition of TRPM1, which in turn produces channel opening and membrane depolarization. In contrast, the absence of TRPM1 leads to membrane hyperpolarization and reduced glutamate release from terminals. A potential problem with this view is that, in the adult mGluR6 KO retina, rod BCs are hyperpolarized and TRPM1 channels are inactive and reduced in number albeit still localized on dendrite tips [15]. However, this observation does not exclude the fact that TRPM1 channels, which are present and active in the mGluR6 KO, can have an intermittent depolarizing effect without mGluR6 during circuit development and even in the adult retina. Rod BCs with small terminal size in the TRPM1 KO retina release less glutamate in response to AC disinhibitory input along the axon passing through the OFF sublamina [31] than those with normal terminal size in the WT or mGluR6 KO retina.

There is a precedent for thinking that subtle differences in the resting membrane potential or activity of rod BCs can lead to noticeable changes in spontaneous activity patterns of RGCs. For example, different mutations affecting the mGluR6 receptor show diverse RGCs activities [32].

In our view, during development, a reduced output either from smaller rod BC terminals or from persistently hyperpolarized rod BCs hyperpolarizes the AII AC network. It is well established that abnormal or absent electrical activity during development can produce abnormal wiring in the visual system [33, 34].

## 5. Conclusion

Mutations of CSNB associated genes, TRPM1 and mGluR6, elicit distinct phenotypes in KO retinas. Our data demonstrate the spontaneous oscillatory firing of RGCs and the smaller size of rod BC terminals in the TRPM1 KO retinas, but not in mGluR6 or WT retinas. Our study assumes that the changes in ON BCs in the TRPM1 KO retina, involving hyperpolarization, reduced transmitter release, or both, lead to a dysregulation of RGC control circuitry that results in oscillations. Future studies should address how the absence of TRPM1 leads to small terminal size, whether a reduction in terminal size is common to all ON BC subtypes, and whether a reduction of terminal size leads to reduced synaptic input and hyperpolarization of AII ACs.
